# Interval Training Is Not Superior to Endurance Training With Respect to Bone Accrual of Ovariectomized Mice

**DOI:** 10.3389/fphys.2020.01096

**Published:** 2020-09-09

**Authors:** Julia Latza, Maresa Otte, Tobias Lindner, Dagmar-Christiane Fischer, Sven Bruhn, Robin Hollinski, Mareike Warkentin, Thomas Mittlmeier, Brigitte Müller-Hilke

**Affiliations:** ^1^Department for Trauma, Hand and Reconstructive Surgery, Rostock University Medical Center, Rostock, Germany; ^2^Core Facility Multimodal Small Animal Imaging, Rostock University Medical Center, Rostock, Germany; ^3^Department of Pediatrics, Rostock University Medical Center, Rostock, Germany; ^4^Department of Exercise Science, Rostock University, Rostock, Germany; ^5^Institute of Diagnostic and Interventional Radiology, Rostock University Medical Center, Rostock, Germany; ^6^Department of Material Science and Medical Engineering, Rostock University, Rostock, Germany; ^7^Core Facility for Cell Sorting and Cell Analysis, Rostock University Medical Center, Rostock, Germany

**Keywords:** osteoporosis, treadmill, bone, micro-computed tomography, mouse, ovariectomy, interval training

## Abstract

Physical exercise is considered to delay bone loss associated with post-menopausal estrogen deficiency in women. However, the optimal training regimen for maximal bone accrual has not yet been defined. We, therefore, turned to ovariectomized (OVX) C57BL/6 mice and directly compared a low intensity endurance training on the treadmill to medium and high intensity interval trainings tailored to the individual performance limits. Trainings lasted 30 min each and were performed five times/week. After a 5-week training period, mice were sacrificed, and the hind legs were analyzed for assessment of (i) biomechanical stability (three-point bending test), (ii) bone microarchitecture [micro-computed tomography (μCT)], (iii) mineral apposition rate (MAR; histomorphometry), and (iv) muscle volume (MRI). Increased running speeds and quadriceps femoris muscle volumes in trained mice confirmed positive impacts on the cardiopulmonary system and myoinduction; however, none of the treadmill training regimens prevented ovariectomy induced bone loss. Our results provide evidence that treadmill training impacts differentially on the various members of the musculoskeletal unit and call for further experiments investigating frequency and duration of training regimens.

## Introduction

Osteoporosis is a major health care problem that is characterized by a loss of bone mass that can lead to pain, immobility, and fragility fractures that, in turn, result in increased mortality (WHO Scientific Group Meeting on Prevention and Management of Osteoporosis, 2004). Osteoporosis is primarily a disease of the elderly; in six European countries (Italy, Spain, Sweden, France, Germany, and UK), a total of 16 million women and 4 million men suffered from osteoporosis in 2015 (International Osteoporosis Foundation, 2019). Approximately 3.2% of women over the age of 50 are affected by fragility fractures every year. The lifetime risk at the age of 50 of experiencing a hip fracture is even higher: up to 22.8% for women and up to 13.7% for men (International Osteoporosis Foundation, 2019). Owing to the demographic changes in the developed countries, absolute numbers of patients and fragility fractures will rise even further. As of yet, there is no cure for osteoporosis, and the most frequent and cost efficient pharmacological treatments include hormone replacement or inhibition of osteoclast activity *via* bisphosphonates (Tella and Gallagher, 2014). However, there are adverse events that, in the case of continuous bisphosphonate uptake, are associated with atypical femoral fractures or osteonecrosis of the jaw bones (Khan, 2008; Chatterjee, 2013).

As mechanical load has been shown to stimulate bone remodeling, physical exercise has frequently been investigated for its benefit on the bone (Moreira et al., 2014; Sumida et al., 2014; Oh et al., 2016). And even though high-impact (e.g., gymnastics, judo, karate, or volleyball) and odd-impact (e.g., soccer, basketball, and speed-skating) training programs suggested the greatest bone accrual for humans, the exact recommendations with respect to quality (running/nordic walking/swimming) and quantity (frequency/duration/intensity) have not yet been defined (Tenforde and Fredericson, 2011; Weidauer et al., 2014; Abrahin et al., 2016; Wang et al., 2016). To circumvent the slow progress of human osteoporosis and the manifold confounding factors associated with human studies, we here turned to a mouse model in order to compare various treadmill training regimens for their propensity to prevent osteoporosis-associated bone loss.

Consensus seems to exist that rodents can model the human skeleton (Frost and Jee, 1992). There is a limitation though because quadrupedal animals – in contrast to humans – experience greater mechanical loading on the appendicular than on the axial bones (Iwamoto et al., 2005). Effects of running exercise on the vertebral bodies of rodents can therefore not be transferred to humans.

Apart from that, the literature on the effect of treadmill training on murine bone shows mixed findings. For example, short term and moderate training periods of 3 weeks led to significantly increased tibial cortical area and periosteal perimeter in male C57BL/6 mice while 8 weeks of a strenuous sprint interval training in the same strain and sex resulted in the significant reduction of cortical bone mass (Wallace et al., 2007; Hollinski et al., 2008). These findings are difficult to reconcile with the hypothesis that bone accrual can be achieved by increasing the frequency and intensity of the impact (Robling and Turner, 2009). Furthermore, recent evidence suggested that male mice may benefit from moderate training regimen while females may require more strain for efficient bone accrual (Wallace et al., 2007; Hollinski et al., 2008; Koenen et al., 2017). All of these studies used different mouse strains, different sexes, and different training regimens, suggesting that firm comparisons can only be drawn from identical experimental set-ups.

We here set out to investigate different training intensities and decided on the model of ovariectomized (OVX) females, as a previous study suggested that OVX within a short time frame led to bone loss, which could partially be prevented by a moderate treadmill exercise (Wu et al., 2001). Performing treadmill training not only allows for axial loading, but also for differential energy conditions as low and medium intensities guarantee aerobic conditions while high intensity trainings that were tailored to the individual performance limits aim at an anaerobic state (Alizadeh et al., 2015). We here concentrated on C57BL/6 females for their superior endurance capacity and the availability of previous publications (Wallace et al., 2007, 2009; Banu et al., 2008; Hollinski et al., 2008; Kvedaras et al., 2017).

## Materials and Methods

### Mice and Experimental Design

**Figure 1:**
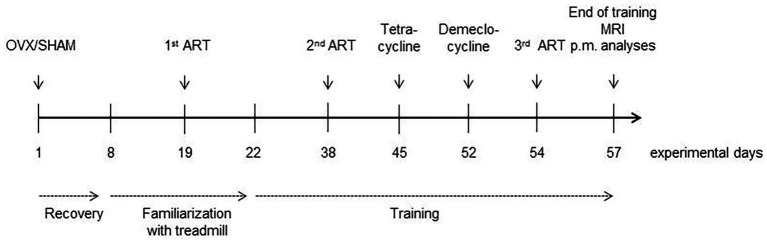
Study design and timeline (time in days). OVX, ovariectomy; SHAM, sham-operation; Familiarization, 2 weeks of familiarization with the treadmill; ART, augmentation run test; ip, injection of tetracycline and demeclocycline intraperitoneal; pm analyses, start of post mortem analyses.

A total of 40 7–8-week old C57BL/6J female mice were purchased from Charles River (Charles River Laboratories, Research Models and Services, Sulzfeld, Germany). In our facility and throughout the experiments, all animals had free access to pellet food and water. They were housed in cages in small groups up to four individuals under a 12/12 h light/dark cycle. After a week of acclimatization, the mice were randomized into five groups of equal numbers (*n* = 8). The first group (I) was sham-operated and served as a control while the other four (II–V) were ovariectomized. After 1 week of recovery, mice in the groups III–V started their 2 weeks familiarization to the treadmill. At the end of this period, all mice accustomed to the treadmill (groups III–V) took part in an augmentation run test (ART) that served to determine the individual maximum velocity (V_max_). For the next 5 weeks, mice in groups III–V were trained on the treadmill, and the ART was repeated twice (after 2.5 weeks and at the end) in order to monitor training effects as changes in the individual V_max_ and to adapt the training accordingly as running speed can be used to tailor training intensities (Høydal et al., 2007). Nine and two days before sacrificing the mice, tetracycline and demeclocycline, respectively, were injected intraperitoneally for subsequent assessment of mineral apposition rate (MAR). At the end of the training period, the volumes of the quadriceps femores’ muscles were measured using 7-Tesla magnetic resonance imaging (MRI). Thereafter, all mice were sacrificed, blood was aspirated, uteri were prepared, and femora and tibiae were preserved. *In-vivo* blood samples could not be taken without weakening the mice and thus influencing the study’s course. An overview of the study design is shown in Figure 1. The local state’s animal care committee (Landesamt für Landwirtschaft, Lebensmittelsicherheit und Fischerei M-V; www.lallf.de) approved all experiments (7221.3-1-021/17), and all experiments were carried out in accordance with the relevant guidelines and regulations.

### Ovariectomy

All animals were anesthetized with ketamine/xylazine (100 mg ketamine and 5 mg xylazine per kg body weight) and kept at 36°C on a warming plate. An ointment was applied to the eyes to prevent dehydration. The lower back was shaved, and a bilateral cut was placed into the skin and peritoneum. The skin of sham-operated mice was re-sutured, while mice of the groups II–V were ovariectomized *via* open exposure: preparation of the uterus, ovaric tube, and ovary, then cauterization of the ovaric tube, and excision of the ovary. All wounds were sutured. For post-operative pain relief, metamizole was added to the drinking water for 5 days.

### Augmentation Run Test

The ART served to determine the individual V_max_ of mice in groups III–V (Hollinski et al., 2008). The treadmill started with a speed of 0.17 m/s for 3 min. It thereafter accelerated over a period of 2 min to 0.2 m/s. This speed was kept for another 3 min before it accelerated again over a period of 2 min to 0.25 m/s. This cycle of constant speed for 3 min and accelerating over a period of 2 min to a speed 0.05 m/s faster than before was maintained until mice no longer kept pace with the treadmill belt and were swept onto the platform behind the running belt. The highest speeds achieved without any external influence represented the individual V_max_.

### Training Modalities

**Figure 2:**
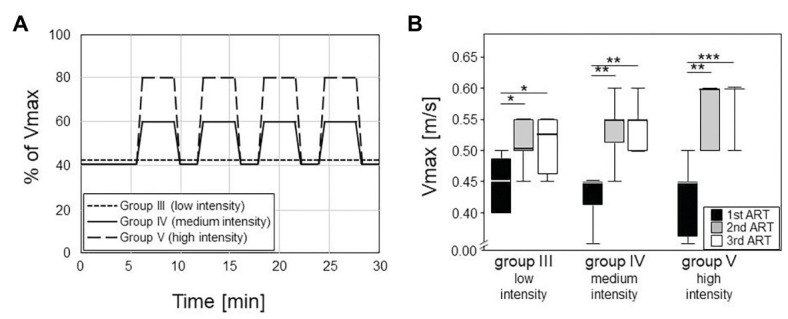
Left panel **(A)** shows the different training modalities for trained groups III – V. The training phase lasted five weeks with five sessions per week, 30 min each (plotted on the horizontal axis). Group III did low intensity training with constant velocity at a 10° incline. Groups IV and V did an interval training with peak intensities of 60% or 80% of the maximum velocity (V_max_, plotted on the vertical axis) and active recovery at 40% of the V_max_. Box plots on the right **(B)** consist of boxes that represent 25th to 75th percentiles, the medians and bars that represent the 5th and 95th percentiles of the data. Right panel shows the differences in V_max_ between the ARTs in all the groups (statistics are calculated with Kruskal-Wallis Test with post-test). From first to second ART, all groups increased their V_max_ significantly (III: *p* = 0.008; IV, V: *p* < 0.001). From second to third ART, none of the groups increased their V_max_ significantly.

Training was performed daily from Monday to Friday, always in the afternoon, and sessions lasted for 30 min. Training for group III consisted of a low intensity endurance run at a constant speed of 0.2 m/s and a 10° incline. Trainings for groups IV and V consisted of medium and high intensity interval trainings without any inclination, respectively. In that regard, mice started at 40% of their V_max_ for 6 min, followed by four repeats of medium (60% of V_max_) or high (80% of V_max_) speed for 3 min interspersed by 1.5 min of recovery at 40% of V_max_ (Figure 2A).

### Magnetic Resonance Imaging

**Figure 3:**
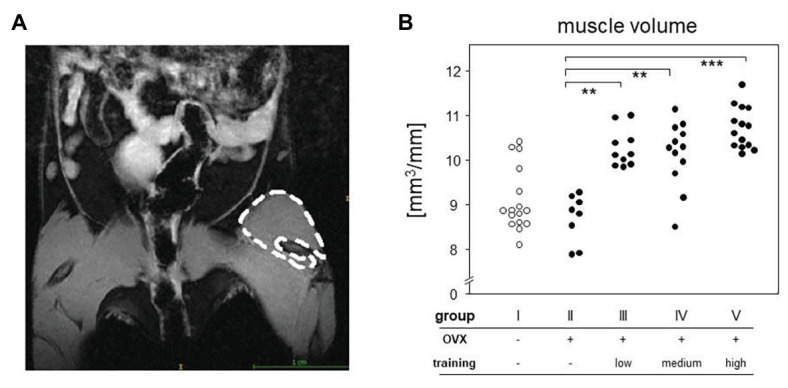
MRI measurement served to quantify the differences between groups. Left picture **(A)** shows an exemplary T2-sequence of abdomen and hind limbs. Left quadriceps femoris muscle of the mouse is marked. The right panel **(B)** represents the muscle volumes of all groups normalized to the respective femoral length. Comparisons were performed *via* Kruskal-Wallis test with post-test (*p* < 0.0001).

Magnetic resonance imaging was used to quantify the volumes of the quadriceps femores muscles. As the muscle volume also depends on length, the ratio of total volume to femur length [calculated as the numbers of micro-computed tomography (μCT) image slices including parts of the femur] was used for statistical analysis. MRI was carried out on a 7-Tesla BioSpec 70/30 (Bruker, Billerica, MA, United States) using a T2 weighted Turbo Rapid Acquisition with Relaxation Enhancement (TurboRARE) sequence with the following settings: TE/TR 25.25/3227 ms, field of view: 28 × 21 mm, matrix size 233 × 175, slice thickness 0.85 mm, resolution 120 × 120 μm, and RARE-factor: 8, capacity spool 72 mm. Mice were anesthetized with isoflurane and were placed on the back with the hind legs fixed. T2 sequence of both hind legs was run. Contours of the quadriceps femores muscles were redrawn manually (Figure 3A), and resulted surfaces were totaled to volumes automatically using ITK-SNAP software (ITK-SNAP 3.4.c 70/30).

### Histomorphometry

For determination of the MAR, left tibiae were fixed in 4% PFA, transferred into ethanol, and stored in the dark before embedding into methyl methacrylate (MMA). Embedding, preparation and laser microtomy were implemented by LLS ROWIAK LaserLabSolutions GmbH (Hannover). A decalcification was not necessary for their TissueSurgeon laser, so structures like growth lines could be analyzed without overlay effects. Furthermore, the contact free cutting caused fewer artifacts. With a cutting speed of approximately 1 mm^2^/s and a diameter of the laser pulse of approximately 1–5 μm, a cutting thickness of 10–100 μm could be accomplished. TissueSurgeon worked with a near infrared laser (1,030 nm). We used fluorochromes with shorter wavelengths, so they were not bleached by the laser.

The sections of 10 μm thickness were then analyzed with a fluorescence microscope (Leica CTR 4000) at the Department of Pediatrics (Rostock University Medical Center). Received data were processed with Leica LAS AF Lite software (Leica Microsystems). Therefore, at 50 different locations within the proximal tibiae, the distance between the two labels (tetracycline and domeclocycline) was measured, displaying the MAR per week (Fischer et al., 2011; Neuerburg et al., 2019).

### X-Ray Micro-Computed Tomography

Cortical and trabecular bone parameters were analyzed using a SkyScan 1076 *In-Vivo* μCT (Bruker, Billerica, MA, United States; Antwerp, Belgium, Software Version 4.2, 0.5 mm Al-filter, integration time of 1.5 s, isotropic voxel size of 9 μm at 49 kV and 200 μA, rotation step of 0.5°, and averaging frame of 3). To that extent, the right femora and tibiae were fixed in 4% PFA, preserved in ethanol. The samples were washed three times in distilled water and equilibrated with the scanning medium, 0.9% saline solution, for at least 12 h for rehydration before measurements. The specimens were placed into the μCT surrounded by a cylindrical polystyrene tube with styrofoam bedding and aligned with the vertical axis of the scanner. At every run, one Eppendorf tube filled with two calcium hydroxide apatite bone mineral density (BMD) calibration rods with densities of 0.25 and 0.75 g/cm^3^ for calibration was supplemented. For reducing artifacts, it was scanned for a full 360°.

The images were processed with NRecon reconstruction software (Bruker, Billerica, MA, United States), DataViewer (Bruker, Billerica, MA, United States), and CT Analyser (Bruker, Billerica, MA, United States). The μCT images were first processed by NRecon software using a Gaussian filter with a smoothing kernel of 2, a ring artifact reduction of 6, a beam hardening correction of 30%, and a defect pixel masking of less than 20%. Afterward, the bones were vertically orientated and rotated in the same way by using DataViewer software. Finally, image analysis was done by CT Analyser software using a global threshold of 64–255 and manufacturers 2D and 3D algorithms for the analyses of cortical and trabecular bone, respectively. The regions of interest (ROIs) for image analyses were determined between two reference levels: For the femora, a lower reference level was set at the distal metaphyseal growth plate and the upper one at the fusion zone of greater trochanter and the femoral head. In case of the tibia, the reference levels were defined between the tibia-fibular syndesmosis (lower reference level) and the proximal epiphyseal growth plate (upper reference level). For both femora and tibiae, the cortical bone was analyzed by defining 10 intervals (ROIs) of 10% each within the upper and lower reference levels. For tibia, we complemented one ROI of identical size below the lower reference level. The segmentation of trabecular and cortical bone regions was performed using automated algorithms, and all ROI interpolations were checked visually before further analysis.

Every ROI in tibia and femora was assayed for cortical area fraction (Ct.Ar/Tt.Ar) and cortical thickness (Ct.Th). Trabecular bone analyses at the femoral metaphyses contained the most distal and proximal 20% within the reference levels. Same rules applied for tibia analyses, just complemented by the lowest ROI up to an interval of 30%. For evaluation of trabecular bone microarchitecture, bone volume fraction (BV/TV), trabecular number (Tb.N), trabecular thickness (Tb.Th), and trabecular bone mineral density (Tb.BMD) were calculated based on 3D calculations (Bouxsein et al., 2010; Behrendt et al., 2016). The calibration for Tb.BMD measurements was performed with the aforementioned phantom rods, which were scanned and reconstructed as described above. The attenuation coefficient was entered into the calibration dialogue window in CT Analyser for determination of Tb.BMD.

### Three-Point Bending Test

Left femora were wrapped in gauze moistened with 0.9% sodium chloride solution and were frozen at −20°C. Before the testing, the bones were thawed at room temperature and then positioned with the posterior condyles downward on a three-point bending machine with a 500 N load cell (zwickiLine Z2.5, Zwick GmbH, Ulm, Germany). The tests were executed at the Department of Material Science and Medical Engineering (Rostock University, Germany). The femoral midshaft was placed directly under the testing stamp with a span length of 6 mm. Applying a gradually increasing bending force with 1 mm/min, the femora were loaded to failure (Jepsen et al., 2015). Bending strength (MPa), postyield displacement (mm), maximum load (N), breaking load (N), and Young’s modulus (MPa) were calculated using the recorded load-displacement curves.

### Statistics

Statistical analyses were performed using IBM SPSS Statistics 25 (IBM, NY, United States), and graphs and figures were generated using SigmaPlot 13.0 (Systat Software, CA, United States). Due to small sample sizes, we used non-parametric tests only – Mann-Whitney for the (non-paired) comparisons of two groups and Kruskal-Wallis with *post-hoc* tests for multiple comparisons. Values of *p* lower than 0.05 were considered significant.

## Results

### Treadmill Training Led to Increased Running Speeds and Accumulated Muscle Volume

Five weeks of treadmill training at a frequency of five times per week were well-accepted, and all mice were developed physiologically over the experimental period. They all gained weight continuously, and this weight gain was independent of the various training modalities. Importantly, this exercise period showed significant training effects as documented 2 folds:

- The first 2.5 weeks led to significant increases in the maximum running speeds (V_max_) as monitored *via* ARTs. In the first ART, all groups had median V_max_ of 0.45 m/s, confirming comparable fitness at the onset of the experiments (Figure 2). Already at the second ART, V_max_ had increased to medians of 0.53 m/s in the low intensity endurance group (group III) and to 0.55 and 0.6 m/s in the medium and high intensity interval training groups (groups IV and V), respectively. The intensities of the trainings were thus reflected in the increases of running speeds. However, running speeds more or less plateaued after the first 2.5 weeks of training. There was hardly any additional increase between the second and third ARTs for groups III and IV while all but one mouse in the high intensity group V reached a V_max_ of 0.6 m/s (Figure 2B).
- Around 5 weeks of continuous treadmill training also resulted in accumulated muscle volume. We here concentrated on the hind legs and the quadriceps femoris muscle and compared its volume among OVX mice that either did or did not participate in the treadmill training. To that extent, we performed MRI and normalized our results to the length of the respective femora. Figure 3 therefore compares areas between the trained groups (groups III–V) with means of 10.61 mm^2^ (group III), 10.41 mm^2^ (group IV), 10.74 mm^2^ (group V), and the non-trained controls (group II) with means of 8.99 mm^2^ (group II), respectively. Of note, there were no differences between the various training regimens. In summary, we here showed that 5 weeks of continuous treadmill exercise resulted in significant training effects with respect to muscle volume and running speeds.

### Treadmill Training Did Not Prevent OVX Induced Bone Loss

**Figure 4:**
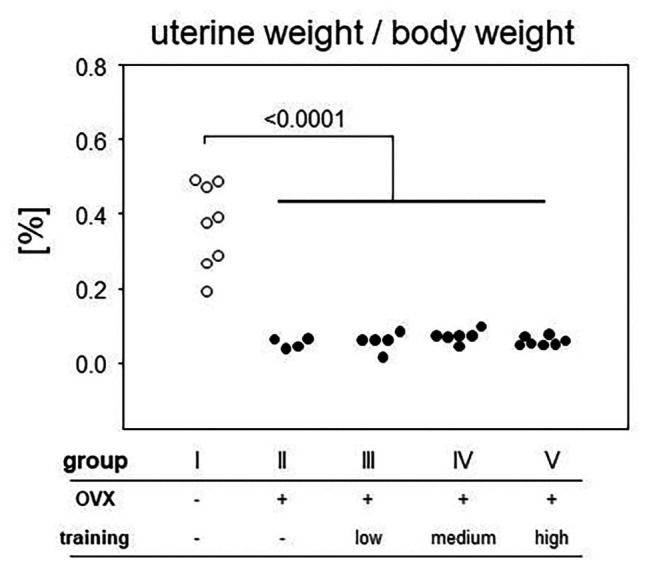
After preparation every uterus was weighed and normalized to the respective body weight. The ratio is plotted on the vertical, experimental groups on the horizontal axis. Statistics are calculated *via* Mann-Whitney test. Dot plots show a significant decrease of uterine weight after ovariectomized (OVX; *p* < 0.0001; group I: sham, group II: OVX, group III: OVX + low intensity training, group IV: OVX + medium intensity training, group V: OVX + high intensity training).

In order to control effective ovariectomy, we analyzed the uteri at the end of the experimental period. Not only were the uteri of OVX mice (groups II–V) less vascularized, they were also significantly smaller as measured by weight (Figure 4). From here on, we concentrated on those mice only that presented a sufficient degeneration of the uteri and therefore excluded nine OVX individuals from further analyses. In detail, three mice were excluded from group II (OVX, no training), three mice from group III (OVX, low intensity training), two from group IV (OVX, medium intensity training), and one from group V (OVX, high intensity training). While the mean uterus weight was 0.37% of the body weight in the sham-operated control mice, OVX resulted in mean relative weights of 0.059%, confirming sustained estrogen-deficiency.

**Figure 5:**
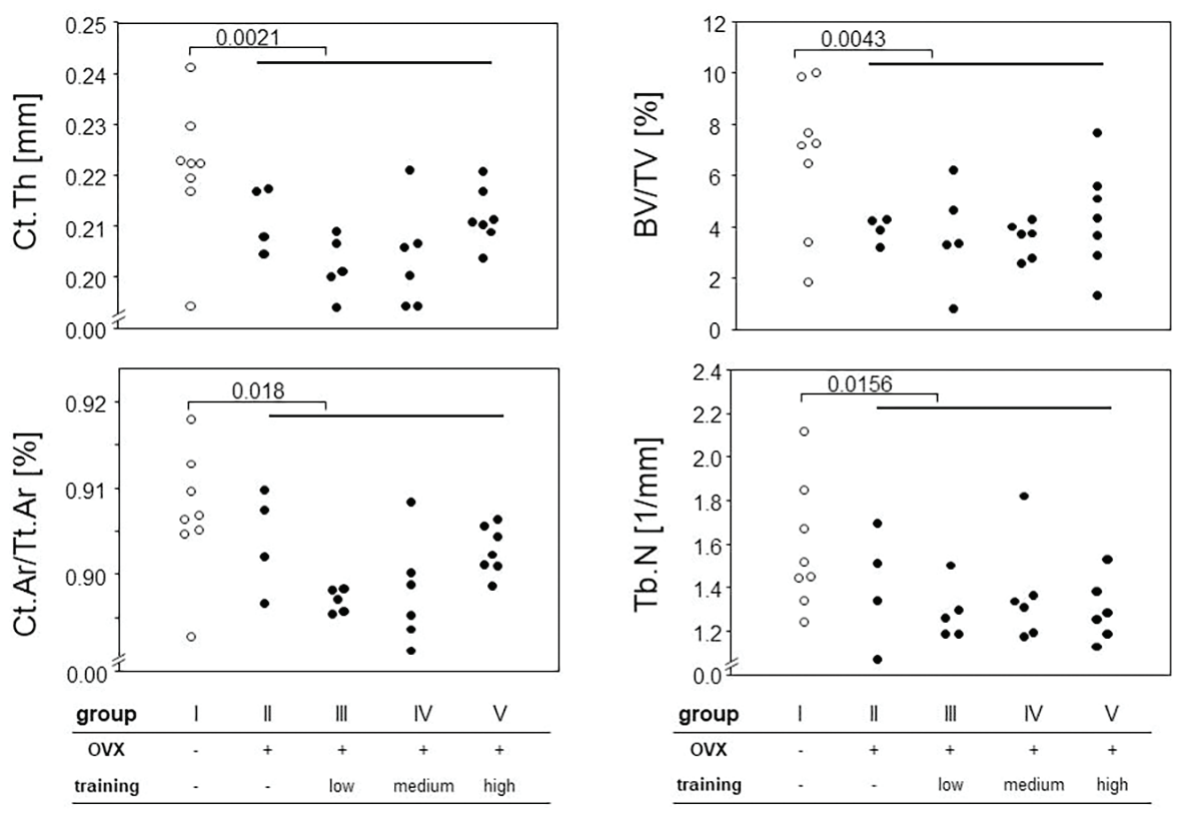
Hind legs were prepared for micro-computed tomography (μCT) measurements. Different parameters for cortical and trabecular bones were calculated (Ct.Th, cortical thickness, Ct.Ar/Tt.Ar, cortical area fraction, BV/TV, bone volume fraction, and Tb.N, trabecular number). **Left panels** represent cortical parameters measured at the femoral diaphysis, and **right panels** trabecular parameters examined at the proximal femur. Surgery and training modalities are indicated in the table below. Comparisons between sham and the OVX-groups were performed *via* Mann-Whitney tests.

We next assessed the impact of OVX on changes to the cortical and trabecular bones. To that extent, we performed μCT of femora and tibiae. While trabecular bone can only reliably be quantified at the distal and proximal ends of these long bones, this is different for the cortical shell. However, as we were curious whether potential cortical changes would occur evenly distributed over the entire length of the long bones, we divided the femora into 10 and the tibiae into 11 equally sized sections that were analyzed individually. Indeed, we observed the most pronounced changes for the cortical bone in the distal parts of the tibiae and the diaphyseal parts of the femora. For the trabecular bone, the effects were very prominent for the proximal parts of both femora and tibiae. Example results are presented in Figure 5 for the diaphyseal femoral region (10% of the midshaft) and the most proximal femoral 20% section. As expected, OVX resulted in a significant loss of cortical and trabecular bones as shown by the comparison between groups I and II–V for Ct.Th, Ct.Ar/Tt.Ar, BV/TV, and Tb.N (Figure 5). While the loss of BV/TV could mostly be attributed to a loss of trabecular numbers, a reduction in cortical bone area (Ct.Ar) and total cross-sectional area (Tt.Ar) indicated periosteal bone loss yet did not allow for conclusions at the endosteum. At proximal and distal regions, Tb.Th stayed unaffected by the ovariectomy while Tb.BMD showed a significant decrease in the groups II–V compared to group I in both tibiae and femora. In summary, OVX led to a significant loss of both cortical and trabecular bones, and none of our various training regimens was capable of preventing or reverting this loss.

### Treadmill Training Decreased Bending Strength

**Figure 6:**
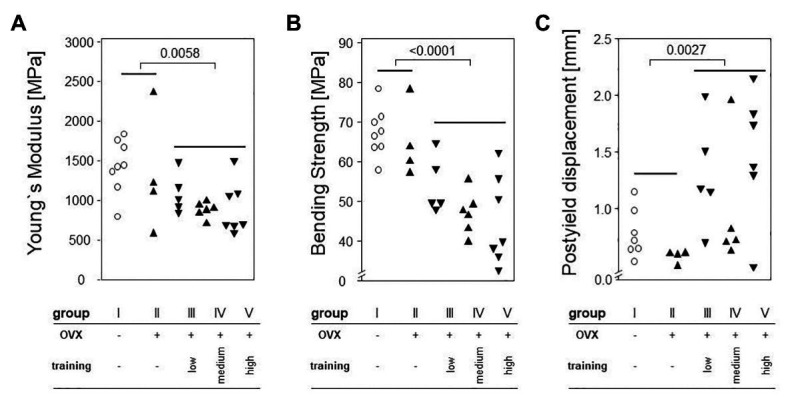
Left femora underwent a three point bending test to quantify the bone’s mechanical stability. Dot plots show comparisons between trained and non-trained groups calculated via Mann-Whitney tests. **(A)** shows Young`s Modulus. **(B)** demonstrates Bending Strength. **(C)** presents Postyield displacement. Statistical significance is indicated be the corresponding *p*-values.

To determine the effects of OVX and treadmill training on the bone’s mechanic stability, three point bending tests were carried out. Neither maximum load, breaking load, bending strength, nor post yield displacement were significantly impacted by OVX, as shown in Figure 6. However, treadmill training had a significant negative impact on the Young’s modulus (Figure 6A) and on bending strength (Figure 6B). Accordingly, the post yield displacement increased significantly (Figure 6C). Of note, there were no significant differences between the various training regimens.

### Ovariectomy but Not the Treadmill Training Impacted on the Mineral Apposition Rate

**Figure 7:**
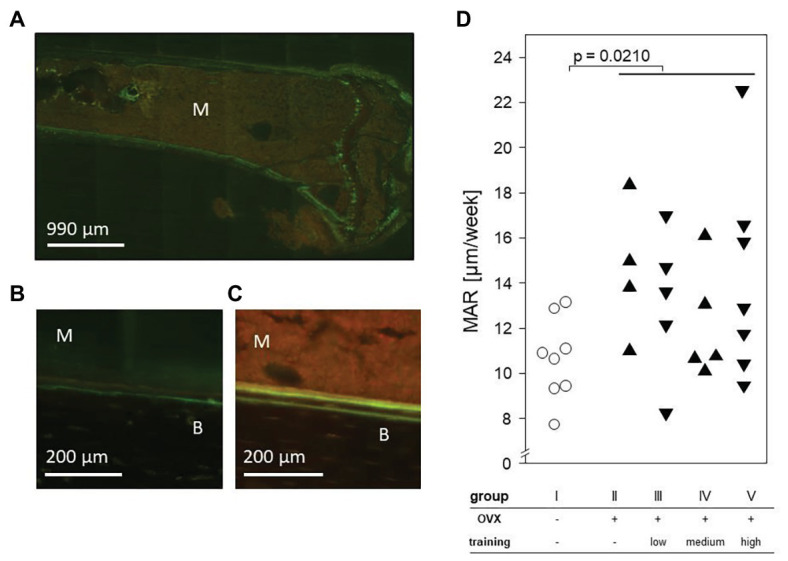
An exemplary fluorescence microscopy of the proximal left tibia after intraperitoneal injection of tetracycline and demeclocycline. **(A)** 25× enlargement. **(B)** + **(C)** 100× enlargement of group I **(B)** and group II **(C)**. The fluorescence labels separate bone **(B)** from marrow (M). **(D)** Dot plot shows the mineral apposition rate (MAR) per week and compares non-OVX to OVX groups *via* Mann-Whitney test. Statistical significance is indicated by the value of *p*.

To determine the impact of ovariectomy and treadmill training on the MAR, tetracycline and demeclocycine were injected 9 and 2 days before sacrificing the animals. Histomorphometric analyses of tibial laser sections revealed the fluorescent label near the endosteum. Importantly, significant increases of MAR were found in all OVX groups (II–V) and resulted in a mean of 13.51 μm/week compared to a mean of 10.61 μm/week in the control mice. However, among the OVX groups, there were no differences between trained and non-trained mice nor among the various training regimens (Figure 7).

## Discussion

We here investigated treadmill running for its potential to prevent or delay OVX induced bone loss in C57BL/6 mice. In detail, we compared three different training regimens, a moderate endurance as well as medium and high intensity interval trainings, and indeed, the main purpose of this study was to identify the most adequate strain for maximal bone accrual. Our experimental design was based on the two assumptions that (i) increased mechanical loads would lead to periosteal bone growth as well as mechanically more efficient structures and that (ii) higher stresses would result in more bone formation (Robling and Turner, 2009). However, while increased running speeds and accumulated muscle volume of the hind legs confirmed positive impacts on the cardiopulmonary system and myoinduction, none of the treadmill training regimens prevented OVX induced bone loss nor did they improve the biomechanical properties of the bone.

The deterioration of biomechanical properties could be attributed to OVX, and this was in line with previous publications (Lambers et al., 2012; Zhang et al., 2016). Likewise, an enhanced MAR at the endosteum as a consequence of OVX has been reported before (Wu et al., 2001). However, we could not repeat previous reports on mice and rats alike that demonstrated bone accrual in response to treadmill training (Iwamoto et al., 1998; Wu et al., 2001; Hamrick et al., 2006; Wallace et al., 2009). Individual results are difficult to compare though, as the published studies not only differ with respect to animal species (rats vs. mice) but also to strain, age, sex, nutritional, and hormonal statuses. While our results are disappointing at first sight, there are several implications that are worth pointing-out.

- Our μCT data suggested that even though treadmill running did not prevent OVX induced bone loss, more Ct.Th and Ct.Ar/Tt.Ar were preserved under more strenuous training regimens (Figure 5). These results are supportive of previous publications showing that high and odd impacts lead to the greatest bone accrual as our interval training included acceleration and deceleration as irregular impacts (Tenforde and Fredericson, 2011; Weidauer et al., 2014). It is along these lines that we would like to address the conflict between our results and those by Wu et al. (2001). They established exercise regimens similar to our moderate endurance training yet used a different mouse strain and performed daily exercise. Strain specific genetics as well as frequencies may therefore play a role.
- Our μCT data further suggested that even though OVX decreased the trabecular bone mass, treadmill training had no additional impact, neither positive nor negative. These observations are in line with previous publications, showing a lack of exercise-associated changes at the proximal tibia and the vertebral bodies alike (Iwamoto et al., 1999; Bennell et al., 2002; Hollinski et al., 2008). This distinctive feature of the trabecular bone is difficult to explain and has in the past been interpreted as a consequence from females accumulating trabecular bone mass in excess of the optimal bone mass, in order to provide a buffer against the bone loss during lactation (Bennell et al., 2002). It may therefore turn out worthwhile to concentrate on the cortical bone only when optimizing a training regimen (Holzer et al., 2009).
- We and others have evidence that longer training periods may be required before a true remodeling and bone accrual can be detected (Hollinski et al., 2008; Hsu et al., 2018). We have previously shown that while 4 weeks of high intensity interval training in young and healthy mice led to a reduction of the tibial cortical thickness, another 4 weeks of training reversed this finding (Hollinski et al., 2008). Likewise, Hsu et al. (2018) showed the upregulation of osteogenesis-related genes in osteoblasts of OVX C57BL/6 mice after 8 weeks of training. We therefore ponder a biphasic response of the bone to increased strain, which in its initial phase is characterized by prevailing osteoclast activity as a means to remove microfissures and damages resulting from excessive stress. And the bone will only adjust to increased demands after some delay and will then shift toward enhanced osteoblast activity and net bone accrual. Along these lines, numerous studies from the rat do not provide any evidence that longer training periods may be detrimental to the bone (Yeh et al., 1993; Iwamoto et al., 1999; Bennell et al., 2002). The exact kinetics still need to be investigated as is the question of whether or not the physiological bone mass measured in non-OVX controls can be exceeded by treadmill training. However, a clear correlation between strain gradients and levels of bone formation has been reported, and future training regimen aiming at maximal bone formation will require a careful adjustment (Stadelmann et al., 2015).

As it was recently reported that the bones of the hind legs respond better to treadmill training even though the fore legs experience higher external forces, we are confident that we did not miss-out positive responses by concentrating on the hind legs only (Wallace et al., 2015). Nonetheless, there are limitations to our study, and these include the rather small numbers of animals that were calculated based on large effect sizes (Wu et al., 2001). Furthermore, there was no control for total training volume but a control for the amount of time by comparing different training intensities. The total training volume was bound to be different in terms of intensities and distances run. Moreover, taking serum samples throughout the training period would have allowed to monitor systemic parameters, indicating bone formation and resorption. Assuming that bones need to adapt to increased strain and that an initial resorption merges with a subsequent formation phase, serum parameters would allow to detect metabolic changes to the bone before results can be quantified *via* imaging.

In summary, our results provide evidence that treadmill training impacts differentially on the various members of the musculoskeletal unit and call for follow-up experiments that will identify training regimens as well as factors that will benefit muscles and bones alike.

## Data Availability Statement

The raw data supporting the conclusions of this article will be made available by the authors, without undue reservation.

## Ethics Statement

The animal study was reviewed and approved by Landesamt für Landwirtschaft, Lebensmittelsicherheit und Fischerei M-V; www.lallf.de (7221.3-1-021/17).

## Author Contributions

TM, BM-H, SB, and D-CF contributed to the conception and design of this study. MW, TL, and RH contributed to the experimental work. MO and JL contributed most to the experimental work. TM is guarantor. BM-H drafted the article. All authors contributed to the analysis of the data. All authors contributed to the critical revision of the article and approved the final manuscript for publication.

### Conflict of Interest

The authors declare that the research was conducted in the absence of any commercial or financial relationships that could be construed as a potential conflict of interest.

## References

[ref1] Abrahin O. Rodrigues R. P. Marçal A. C. Alves E. A. C. Figueiredo R. C. de Sousa E. C. (2016). Swimming and cycling do not cause positive effects on bone mineral density: a systematic review. Rev. Bras. Reumatol. 56, 345–351. 10.1016/j.rbr.2015.09.010, PMID: 27476628

[ref2] Alizadeh H. Daryanoosh F. Moatari M. Hoseinzadeh K. (2015). Effects of aerobic and anaerobic training programs together with omega-3 supplement on interleukin-17 and CRP plasma levels in male mice. Med. J. Islam Repub. Iran 29:236. PMID: 26793627 PMC4715379

[ref3] Banu J. Bhattacharya A. Rahman M. Fernandes G. (2008). Beneficial effects of conjugated linoleic acid and exercise on bone of middle-aged female mice. J. Bone Miner. Metab. 26, 436–445. 10.1007/s00774-008-0863-3, PMID: 18758901

[ref4] Behrendt A. -K. Kuhla A. Osterberg A. Polley C. Herlyn P. Fischer D. -C.. (2016). Dietary restriction-induced alterations in bone phenotype: effects of lifelong versus short-term caloric restriction on femoral and vertebral bone in C57BL/6 mice. J. Bone Miner. Res. 31, 852–863. 10.1002/jbmr.2745, PMID: 26572927

[ref5] Bennell K. L. Khan K. M. Warmington S. Forwood M. R. Coleman B. D. Bennett M. B.. (2002). Age does not influence the bone response to treadmill exercise in female rats. Med. Sci. Sports Exerc. 34, 1958–1965. 10.1097/00005768-200212000-00015, PMID: 12471302

[ref6] Bouxsein M. L. Boyd S. K. Christiansen B. A. Guldberg R. E. Jepsen K. J. Müller R. (2010). Guidelines for assessment of bone microstructure in rodents using micro-computed tomography. J. Bone Miner. Res. 25, 1468–1486. 10.1002/jbmr.141, PMID: 20533309

[ref7] Chatterjee S. (2013). Atypical femoral fractures associated with long-term bisphosphonate use. CMAJ 185:1248. 10.1503/cmaj.121698, PMID: 23649415 PMC3787173

[ref8] Fischer D. -C. Jensen C. Rahn A. Salewski B. Kundt G. Behets G. J.. (2011). Ibandronate affects bone growth and mineralization in rats with normal and reduced renal function. Pediatr. Nephrol. 26, 111–117. 10.1007/s00467-010-1660-5, PMID: 20953634

[ref9] Frost H. M. Jee W. S. S. (1992). On the rat model of human osteopenias and osteoporoses. Bone Miner. 18, 227–236. 10.1016/0169-6009(92)90809-R, PMID: 1392696

[ref10] Hamrick M. W. Skedros J. G. Pennington C. McNeil P. L. (2006). Increased osteogenic response to exercise in metaphyseal versus diaphyseal cortical bone. J. Musculoskelet. Neuronal Interact. 6, 258–263. 17142947

[ref11] Hollinski R. Osterberg A. Polei S. Lindner T. Cantré D. Mittlmeier T.. (2008). Young and healthy C57BL/6 J mice performing sprint interval training reveal gender‐ and site-specific changes to the cortical bone. Sci. Rep. 8:1529. 10.1038/s41598-018-19547-z, PMID: 29367742 PMC5784077

[ref12] Holzer G. Skrbensky G. von Holzer L. A. Pichl W. (2009). Hip fractures and the contribution of cortical versus trabecular bone to femoral neck strength. J. Bone Miner. Res. 24, 468–474. 10.1359/jbmr.081108, PMID: 19016592

[ref13] Høydal M. A. Wisløff U. Kemi O. J. Ellingsen O. (2007). Running speed and maximal oxygen uptake in rats and mice: practical implications for exercise training. Eur. J. Cardiovasc. Prev. Rehabil. 14, 753–760. 10.1097/HJR.0b013e3281eacef1, PMID: 18043295

[ref14] Hsu W. -B. Hsu W. -H. Hung J. -S. Shen W. -J. Hsu R. W. -W. (2018). Transcriptome analysis of osteoblasts in an ovariectomized mouse model in response to physical exercise. Bone Joint Res. 7, 601–608. 10.1302/2046-3758.711.BJR-2018-0075.R2, PMID: 30581558 PMC6269594

[ref15] International Osteoporosis Foundation (2019). IOF Report_EU. Broken Bones, Broken Lives: a roadmap to solve the fragility fracture crisis in Europe. Available at: http://share.iofbonehealth.org/EU-6-Material/Reports/IOF%20Report_EU.pdf (Accessed August 23, 2020).

[ref16] Iwamoto J. Takeda T. Ichimura S. (1998). Effects of moderate intensity exercise on tibial bone mass in mature ovariectomized rats: bone histomorphometry study. Keio J. Med. 47, 162–167. 10.2302/kjm.47.162, PMID: 9785762

[ref17] Iwamoto J. Takeda T. Sato Y. (2005). Effect of treadmill exercise on bone mass in female rats. Exp. Anim. 54, 1–6. 10.1538/expanim.54.1, PMID: 15725675

[ref18] Iwamoto J. Yeh J. K. Aloia J. F. (1999). Differential effect of treadmill exercise on three cancellous bone sites in the young growing rat. Bone 24, 163–169. 10.1016/S8756-3282(98)00189-6, PMID: 10071907

[ref19] Jepsen K. J. Silva M. J. Vashishth D. Guo X. E. van der Meulen M. C. H. (2015). Establishing biomechanical mechanisms in mouse models: practical guidelines for systematically evaluating phenotypic changes in the diaphyses of long bones. J. Bone Miner. Res. 30, 951–966. 10.1002/jbmr.2539, PMID: 25917136 PMC4794979

[ref20] Khan A. (2008). Bisphosphonate-associated osteonecrosis of the jaw. Can. Fam. Physician 54, 1019–1021. PMID: 18625828 PMC2464788

[ref21] Koenen K. Knepper I. Klodt M. Osterberg A. Stratos I. Mittlmeier T.. (2017). Sprint interval training induces a sexual dimorphism but does not improve peak bone mass in Young and healthy mice. Sci. Rep. 7:44047. 10.1038/srep44047, PMID: 28303909 PMC5355982

[ref22] Kvedaras M. Minderis P. Fokin A. Ratkevicius A. Venckunas T. Lionikas A. (2017). Forced running endurance is influenced by gene(s) on mouse chromosome 10. Front. Physiol. 8:9. 10.3389/fphys.2017.00009, PMID: 28167917 PMC5253375

[ref23] Lambers F. M. Kuhn G. Schulte F. A. Koch K. Muller R. (2012). Longitudinal assessment of in vivo bone dynamics in a mouse tail model of postmenopausal osteoporosis. Calcif. Tissue Int. 90, 108–119. 10.1007/s00223-011-9553-6, PMID: 22159822

[ref24] Moreira L. D. F. Oliveira M. L. de Lirani-Galvão A. P. Marin-Mio R. V. Santos R. N. D. Lazaretti-Castro M. (2014). Physical exercise and osteoporosis: effects of different types of exercises on bone and physical function of postmenopausal women. Arq. Bras. Endocrinol. Metabol. 58, 514–522. 10.1590/0004-2730000003374, PMID: 25166042

[ref25] Neuerburg C. Mittlmeier L. M. Keppler A. M. Westphal I. Glass Ä. Saller M. M.. (2019). Growth factor-mediated augmentation of long bones: evaluation of a BMP-7 loaded thermoresponsive hydrogel in a murine femoral intramedullary injection model. J. Orthop. Surg. Res. 14:297. 10.1186/s13018-019-1315-6, PMID: 31488155 PMC6727400

[ref26] Oh T. Tanaka S. Naka T. Igawa S. (2016). Effects of high-intensity swimming training on the bones of ovariectomized rats. J. Exerc. Nutr. Biochem. 20, 39–45. 10.20463/jenb.2016.09.20.3.6, PMID: 27757386 PMC5067419

[ref27] Robling A. G. Turner C. H. (2009). Mechanical signaling for bone modeling and remodeling. Crit. Rev. Eukaryot. Gene Expr. 19, 319–338. 10.1615/CritRevEukarGeneExpr.v19.i4.50, PMID: 19817708 PMC3743123

[ref28] Stadelmann V. A. Brun J. Bonnet N. (2015). Preclinical mouse models for assessing axial compression of long bones during exercise. Bonekey Rep. 4:768. 10.1038/bonekey.2015.138, PMID: 26788286 PMC4704463

[ref29] Sumida S. Iwamoto J. Uenishi K. Otani T. (2014). One-year changes in bone mineral density and bone turnover markers in premenopausal amateur runners: a prospective study. Keio J. Med. 63, 43–51. 10.2302/kjm.2013-0010-OA, PMID: 24920066

[ref30] Tella S. H. Gallagher J. C. (2014). Prevention and treatment of postmenopausal osteoporosis. J. Steroid Biochem. Mol. Biol. 142, 155–170. 10.1016/j.jsbmb.2013.09.008, PMID: 24176761 PMC4187361

[ref31] Tenforde A. S. Fredericson M. (2011). Influence of sports participation on bone health in the young athlete: a review of the literature. PM R 3, 861–867. 10.1016/j.pmrj.2011.05.019, PMID: 21944303

[ref32] Wallace I. J. Pagnotti G. M. Rubin-Sigler J. Naeher M. Copes L. E. Judex S.. (2015). Focal enhancement of the skeleton to exercise correlates with responsivity of bone marrow mesenchymal stem cells rather than peak external forces. J. Exp. Biol. 218, 3002–3009. 10.1242/jeb.118729, PMID: 26232415 PMC4631774

[ref33] Wallace J. M. Rajachar R. M. Allen M. R. Bloomfield S. A. Robey P. G. Young M. F.. (2007). Exercise-induced changes in the cortical bone of growing mice are bone‐ and gender-specific. Bone 40, 1120–1127. 10.1016/j.bone.2006.12.002, PMID: 17240210 PMC2729655

[ref34] Wallace J. M. Ron M. S. Kohn D. H. (2009). Short-term exercise in mice increases tibial post-yield mechanical properties while two weeks of latency following exercise increases tissue-level strength. Calcif. Tissue Int. 84, 297–304. 10.1007/s00223-009-9228-8, PMID: 19283427

[ref35] Wang L. Xu X. Zhang Y. Hao H. Chen L. Su T.. (2016). A model of health education and management for osteoporosis prevention. Exp. Ther. Med. 12, 3797–3805. 10.3892/etm.2016.3822, PMID: 28105113 PMC5228464

[ref36] Weidauer L. Minett M. Negus C. Binkley T. Vukovich M. Wey H.. (2014). Odd-impact loading results in increased cortical area and moments of inertia in collegiate athletes. Eur. J. Appl. Physiol. 114, 1429–1438. 10.1007/s00421-014-2870-5, PMID: 24664495

[ref37] WHO Scientific Group Meeting on Prevention and Management of Osteoporosis (2004). WHO Scientific group on the assessment of osteoporosis at primary health care level. Available at: https://www.who.int/chp/topics/Osteoporosis.pdf (Accessed February 12, 2019).

[ref38] Wu J. Wang X. X. Takasaki M. Ohta A. Higuchi M. Ishimi Y. (2001). Cooperative effects of exercise training and genistein administration on bone mass in ovariectomized mice. J. Bone Miner. Res. 16, 1829–1836. 10.1359/jbmr.2001.16.10.1829, PMID: 11585347

[ref39] Yeh J. K. Aloia J. F. Chen M. M. Tierney J. M. Sprintz S. (1993). Influence of exercise on cancellous bone of the aged female rat. J. Bone Miner. Res. 8, 1117–1125. 10.1002/jbmr.5650080913, PMID: 8237482

[ref40] Zhang Z. Zhang Q. Yang H. Liu W. Zhang N. Qin L.. (2016). Monotropein isolated from the roots of Morinda officinalis increases osteoblastic bone formation and prevents bone loss in ovariectomized mice. Fitoterapia 110, 166–172. 10.1016/j.fitote.2016.03.013, PMID: 26996879

